# An EEG study on the somatotopic organisation of sensorimotor cortex activation during action execution and observation in infancy

**DOI:** 10.1016/j.dcn.2015.08.004

**Published:** 2015-08-17

**Authors:** Carina C.J.M. de Klerk, Mark H. Johnson, Victoria Southgate

**Affiliations:** Centre for Brain and Cognitive Development, Birkbeck College, University of London, United Kingdom

**Keywords:** Somatotopy, Electroencephalography, Sensorimotor alpha, Action perception, Infancy

## Abstract

Previous studies have shown that sensorimotor cortex activation is somatotopically-organised during action execution and observation in adulthood. Here we aimed to investigate the development of this phenomenon in infancy. We elicited arm and leg actions from 12-month-old infants and presented them, and a control group of adults, with videos of arm and leg actions while we measured their sensorimotor alpha suppression using EEG. Sensorimotor alpha suppression during action execution was somatotopically organised in 12-month-old infants: there was more suppression over the arm areas when infants performed reaching actions, and more suppression over the leg area when they performed kicking actions. Adults also showed somatotopically-organised activation during the observation of reaching and kicking actions. In contrast, infants did not show somatotopically-organised activation during action observation, but instead activated the arm areas when observing both reaching and kicking actions. We suggest that the somatotopic organisation of sensorimotor cortex activation during action observation may depend on infants’ understanding of the action goal and their expectations about how this goal will be achieved.

## Highlights

• EEG study on somatotopy of sensorimotor cortex activation in 12-month-old infants.
• Infants showed somatotopically-organised activation during action execution.
• Infants did not show somatotopically-organised activation during action observation.
• Instead, infants activated the arm areas when observing both arm and leg actions.
• Infants may have emulated the goal of the relatively unfamiliar leg actions.

## 1 Introduction

Ever since the discovery of motor neurons that are activated during the observation of others’ actions in both monkeys (Gallese et al., 1996) and humans (Fadiga et al., 1995, Mukamel et al., 2010), there has been a renewed interest in the idea that observing, imagining, or in any way representing an action, activates the motor programmes that are typically used to execute that same action (James, 1890, Jeannerod, 1994, Prinz, 1997, Stock and Stock, 2004). Infants’ limited, yet developing motor repertoire has the potential to shed light on the development of this phenomenon, and in recent years many researchers have investigated the relationship between action execution and action observation in infancy (e.g. Longo and Bertenthal, 2006, Sommerville et al., 2005, van Elk et al., 2008, Virji-Babul et al., 2012, Von Hofsten, 2007). Many of these studies have investigated the shared neural activation during action execution and observation by measuring alpha suppression over the sensorimotor areas using electroencephalography (EEG) (Marshall et al., 2011, Southgate et al., 2009, Southgate et al., 2010, van Elk et al., 2008, Virji-Babul et al., 2012). While at rest, sensorimotor neurons fire spontaneously in synchrony leading to large amplitude EEG oscillations in the alpha frequency band (8–13 Hz in adults and 6–9 Hz in infants) (Pineda, 2005, Stroganova et al., 1999). Whenever the sensorimotor cortex is activated, i.e. during the execution and observation of actions, the firing of the neurons becomes desynchronized leading to a decrease in power of the sensorimotor alpha-band oscillations (Pfurtscheller and Neuper, 1997, Salmelin and Hari, 1994). The sensorimotor alpha rhythm is distinct from the visual alpha rhythm at posterior sites (Stroganova et al., 1999), and is attenuated in response to both the observation and execution of actions from at least 9 months of age (Marshall et al., 2011, Southgate et al., 2009, Southgate et al., 2010). Source localisation analyses of MEG data suggest that the sensorimotor alpha rhythm most likely originates in the somatosensory cortex (Hari and Salmelin, 1997, Salmelin et al., 1995), which has been shown to have mirroring properties (Gazzola and Keysers, 2009) but is not typically considered to be part of the human mirror neuron system (MNS). However, a recent adult study combining EEG and fMRI recordings has demonstrated that sensorimotor alpha suppression correlates significantly with the BOLD signal in motor areas such as inferior parietal lobule and dorsal premotor cortex during action observation and execution (Arnstein et al., 2011). These findings support the notion that sensorimotor alpha suppression reflects the modulation of sensorimotor cortex activation by mirror neuron areas in the parietal and frontal cortex, and suggest that it can be used as a valid indirect index of MNS activity (Arnstein et al., 2011, Hari et al., 1998, Muthukumaraswamy and Johnson, 2004a, Muthukumaraswamy and Johnson, 2004b, Muthukumaraswamy et al., 2004, Nyström et al., 2011, Perry and Bentin, 2009). Together with the relative ease with which EEG can be used with young infants, this has made sensorimotor alpha suppression the most frequently used neural measure of action mirroring in infancy.

**Fig. 1:**
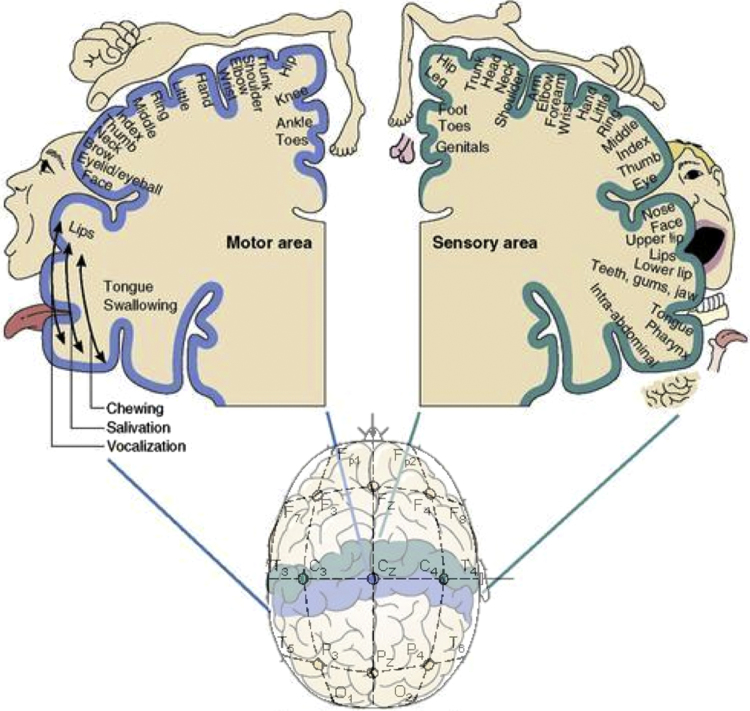
The international 10–20 system of EEG electrode placement overlaid on the somatotopic organisation of the sensorimotor cortex.

The majority of previous infant EEG studies investigating the involvement of the motor system in action perception have measured sensorimotor alpha suppression during the observation of arm actions (e.g. Marshall et al., 2011, Nyström et al., 2011, Paulus et al., 2012, Ruysschaert et al., 2013, Southgate et al., 2009, Southgate et al., 2010, Southgate and Begus, 2013, Southgate and Vernetti, 2014, Stapel et al., 2010, Warreyn et al., 2013). Here we aimed to explore the somatotopic organisation of the sensorimotor cortex in infancy by investigating to what extent different regions of the sensorimotor cortex are recruited during the execution and observation of both arm and leg actions. When we began this work there was only one study in which infants observed leg movements (van Elk et al., 2008). In this study, sensorimotor alpha suppression during the observation of crawling and walking actions was maximal at the Cz electrode overlying the medial leg representation area, while in previous studies alpha suppression during the observation of arm actions had been maximal over the C3 and C4 electrode positions overlying the arm representation areas (e.g. Southgate et al., 2009, Southgate et al., 2010, see also Fig. 1), suggesting that the topography of sensorimotor alpha suppression during the observation of arm and leg actions might be different. However, there was no study that systematically investigated the somatotopic organisation of sensorimotor alpha suppression in infancy (but see recent work by Saby et al. (2013)). Therefore, the present study aimed to compare the scalp distribution of sensorimotor alpha suppression during the execution and observation of arm and leg movements in 12-month-old infants and a control group of adults.

### 1.1 Somatotopic organisation of sensorimotor cortex in adults

Previous work with adult participants has demonstrated that the execution or imagination of specific motor acts results in localised, somatotopically-organised alpha suppression over the sensorimotor cortex (Pfurtscheller et al., 1997, Pfurtscheller et al., 2000, Pfurtscheller et al., 2006). As can be seen in Fig. 1, in the 10–20 system electrode positions C3 and C4 overlie the hand representation area and electrode position Cz overlies the leg representation area of the classical sensory and motor homunculus (Penfield and Rasmussen, 1950). Pfurtscheller and colleagues found that performed or imagined hand actions resulted in more alpha suppression at C3 and C4 overlying the lateral hand representation areas, while performed or imagined foot actions resulted in more suppression at Cz overlying the more medial foot representation areas (Pfurtscheller et al., 1997, Pfurtscheller et al., 2000).

fMRI studies have demonstrated that the *observation* of actions performed with different effectors also results in somatotopically-organised activation of the motor cortex (e.g. Buccino et al., 2001, Hauk et al., 2004, Wheaton et al., 2004). For example, Buccino et al. (2001) found somatopically-organised activation of the premotor and parietal cortex during the observation of foot, hand, and mouth actions. However, no previous adult experiments have used EEG to investigate the somatotopic organisation of sensorimotor cortex activation during action observation. Confirming that sensorimotor alpha suppression during action observation is somatotopically organised in adults therefore was an important first step to validate the measure, and to ensure that the stimuli that were used were able to elicit a somatotopic response.

### 1.2 Development of somatotopic organisation of sensorimotor cortex

A study with premature infants demonstrated that sensory stimulation of the hand and foot resulted in somatotopically-organised oscillatory EEG activity over the somatosensory cortex (Milh et al., 2007). These findings suggest that the somatotopic arrangement of the *somatosensory* cortex in response to sensory stimulation may already develop in utero (Milh et al., 2007). However, it is unclear whether the neural activation during the *execution* of actions, i.e. activation related to the motor part of the sensorimotor cortex, is somatotopically organised at such an early point in development as well. Another unanswered question concerns the development of the somatotopic organisation of sensorimotor alpha suppression during action observation. After the present study was completed, another study was published that also investigated the somatotopic organisation of sensorimotor alpha suppression during action observation in infancy (Saby et al., 2013). In this EEG study, 14-month-old infants observed a live model perform a button-pressing action with either the foot or the hand. Infants who observed the hand actions demonstrated more sensorimotor alpha suppression over the hand areas, while infants who observed the foot actions showed more sensorimotor alpha suppression over the foot area.

### 1.3 The present study

The present study extends this work by investigating the somatotopic organisation of sensorimotor cortex, as measured by sensorimotor alpha suppression, during both action execution and observation in 12-month-old infants. Additionally, a group of adult participants was included to verify whether our stimuli were able to elicit a somatotopic sensorimotor cortex response. Infants and adults were presented with videos of arm (i.e. pushing) and leg (i.e. kicking) actions while their EEG was measured. After the observation phase, infants were encouraged to perform arm and leg movements themselves (i.e. reaching and kicking). Based on the existing studies with adults, infants were expected to show greater sensorimotor alpha suppression over the lateral arm areas when performing reaching actions and greater suppression over the medial leg area when performing kicking actions. We expected to find similar somatotopically organised suppression during the observation of the arm and leg actions in both infants and adults.

## 2 Methods

### 2.1 Participants

The final sample for the observation phase of the study consisted of twenty-seven 12-month-old infants (14 females, mean age = 12 months and 3 days; *M* = 368.2 days, range 351–388 days) and seventeen adults (12 females, mean age = 30 years and 10 months; range 18–48 years). An additional ten adults were tested but excluded because of poor data quality (9) or medical history (1 participant had been in a coma). An additional thirty-nine infants were tested but excluded because they did not provide enough artefact-free trials for analyses due to movement, fussiness, or poor signal quality (37), equipment failure (1), or experimenter error (1). The percentage of excluded infants (59%) is similar to other EEG studies with infants this age (Stapel et al., 2010, van Elk et al., 2008). The final sample for the execution phase of the study consisted of twenty-four 12-month-old infants (13 females, mean age = 12 months and 2 days; *M* = 367 days, range 348–389 days) who also participated in the observation phase of this experiment (*N* = 18) or who performed kicking and reaching actions during the pilot phase of this study (*N* = 6). Too small a proportion of infants contributed a sufficient amount of artefact-free trials in both the observation and execution condition for these two conditions to be directly compared. All infants were born full-term, healthy and with normal birth weight. Ethical approval for this study was obtained from the Birkbeck School of Psychology Ethics committee. Written informed consent was obtained from adult participants and from the infant's caregiver prior to the start of the experiment.

### 2.2 Stimuli

**Fig. 2:**
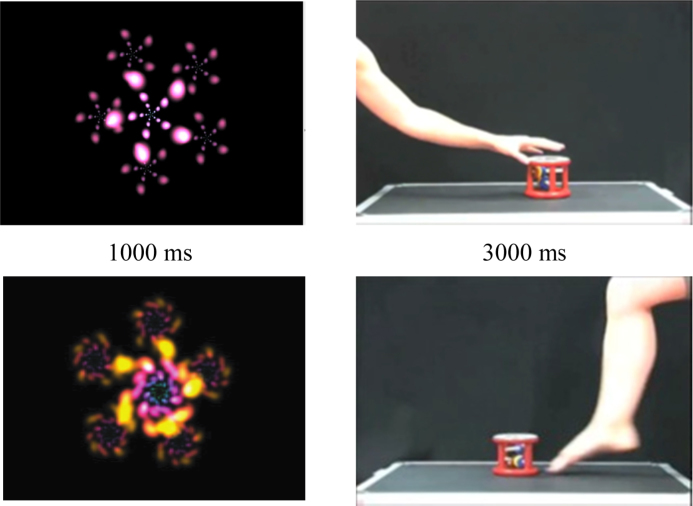
Still frames from the baseline videos and the videos of the arm and leg actions.

The stimulus material consisted of 3-s video clips of simple leg (i.e. kicking) and arm (i.e. pushing) actions performed by an adult model. The actions were shown from a side-view in which only the arm or leg of the model was visible (see Fig. 2). The arm or leg moved towards a colourful toy and either kicked or pushed the toy forward. Thereafter the arm or leg moved back to the starting position. The model performed the actions with her right arm and leg and pushed or kicked the toy to the left side of the screen. The images were flipped to create videos in which a left arm or leg seemed to be pushing or kicking the toy to the right side of the screen. Each experimental trial was 4000 ms and comprised a baseline period (1000 ms) followed by a pushing or kicking action (3000 ms). During the baseline period, a moving screensaver-like image was shown which controlled for activation related to the observation of movement (see Fig. 2).

### 2.3 Procedure

#### 2.3.1 Observation phase

Infants were seated on their caregiver's lap in a darkened room at a distance of approximately 80 cm from a 51-in. plasma screen on which the visual stimuli were presented. Infants were randomly allocated to the ‘Leg’ (*N* = 15) or ‘Arm’ (*N* = 12) condition. The order of left- and right-ward kicking or pushing actions was randomised. The experimenter triggered the presentation of brief attention-getting sounds at random intervals to attract or maintain the infant's attention to the screen. This part of the study lasted up to 8 min or until the infant was no longer willing to watch the stimuli. Adults watched 30 videos of each trial type (‘Arm’ or ‘Leg’ actions) presented in blocks. Which of the two conditions was presented first was counterbalanced between participants, and the order of left- and right-ward kicking or pushing actions was randomised.

#### 2.3.2 Execution phase

**Fig. 3:**
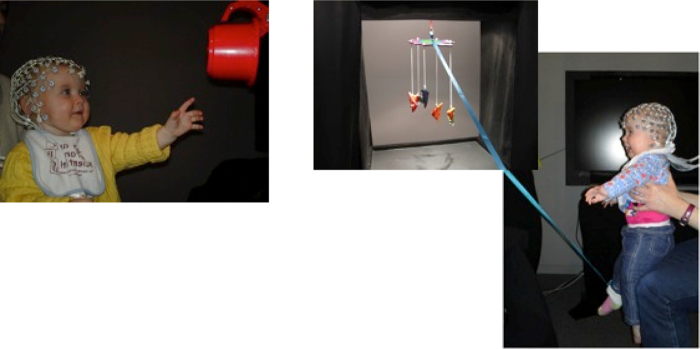
Experimental setup to elicit reaching and kicking actions. A mechanical claw was used to hand graspable toys to the infant. To elicit leg actions a ribbon that was connected to a colourful infant mobile was loosely tied around the ankle of the infant's right foot. The conjugate movement and sound of the mobile reinforced kicking movements.

The execution phase directly followed the observation phase. To elicit arm actions the experimenter waited for the infants to sit still and then handed them a toy using a mechanical claw (Fig. 3). Once the infant reached for and grasped the toy, the experimenter removed the claw. A second experimenter retrieved the toy after allowing the infant to briefly play with it. This procedure was repeated until the infant was bored, or until he/she had reached for approximately 10 different toys. To elicit leg actions a mobile conjugate paradigm (Borovsky and Rovee-Collier, 1990) was used in which a ribbon that was connected to a colourful infant mobile was loosely tied around the ankle of the infant's right foot (see Fig. 3). The mobile had little bells attached to it so that the infant was reinforced to perform kicking movements by the conjugate movement and sound of the mobile. If necessary, one of the experimenters gently moved the infants’ legs to show them that their leg movements caused the mobile to move. EEG was recorded during approximately 8 leg movements or until the infant stopped moving her legs or became fussy. Note that infants performed slightly different actions than that they observed in the observation phase of the study. This was mainly for practical reasons as we expected that not all 12-month-old infants would be able to push a toy away or perform goal-directed kicking actions. We used reaching and mobile kicking actions instead, to maximise our chances of obtaining good action execution data.

### 2.4 EEG processing

EEG was recorded using a 128-electrode Geodesic Sensor Net (EGI Inc., Eugene, Oregon). EEG was recorded with respect to the vertex electrode and re-referenced to the average reference after the artefact detection and rejection process. EEG was recorded by a Net Amps amplifier with a hardware filter between .1 Hz (high-pass) and 100 Hz (low-pass) and a sampling rate of 500 Hz. No offline filtering was performed. EEG data was recorded and pre-processed using NetStation and analysed using WTools (developed by E. Parise, L. Filippin, & G. Csibra, available upon request).

#### 2.4.1 Execution phase

Infants were video recorded throughout the execution phase and trials in which the infants moved both effectors were excluded (i.e. reaching trials in which infants also moved their legs, or kicking trials in which infants also moved their arms). Only trials that were preceded by at least 900 ms during which the infant was sitting still were included. Furthermore, trials with excessive movement artefacts were rejected based on visual inspection. Results of a previous study suggested that very few trials are required to observe sensorimotor alpha suppression during action execution (Southgate et al., 2009), so all infants with at least 2 artefact-free trials were included in the analyses (see Southgate et al., 2009 and Supplementary Fig. 1 in Appendix A for examples of single trial effects). Infants contributed a mean of 3.4 artefact-free kicking (SD = 1.31; range: 2–7 trials) and 4 artefact-free reaching trials (SD = 1.67; range: 2–8 trials) to the analysis. There were too few trials of each type to separately analyse suppression related to the execution of left- or right- kicking or reaching actions, or unilateral versus bilateral reaching and kicking actions. Instead, left and right unilateral and bilateral reaching and kicking actions were all averaged together in the analyses.

EEG data was segmented into 2800 ms trials, consisting of a 1000 ms pre-movement baseline, a 1000 ms analysis period, and a 400 ms buffer on either side of the segment. Time-frequency analyses were performed on each artefact-free trial by continuous wavelet transform using Morlet wavelets at 1 Hz intervals in the 5–25 Hz range. To eliminate distortion created by the wavelet transform, the first and last 400 ms of each trial were removed after the transformation. A 500 ms baseline period, beginning 800 ms before the onset the movement was chosen. Activity in the 6–9 Hz frequency range during this baseline period was subtracted from activity during 500 ms of the analysis period. As sensorimotor alpha suppression was maximal slightly earlier for the kicking, compared to the reaching actions, a different analysis time window was chosen for these two movements: 200–700 ms after movement onset for the kicking and 500–1000 ms after movement onset for the reaching.

#### 2.4.2 Observation phase

Adults and infants were video recorded throughout the session and trials in which they did not attend the screen or made any limb movements were excluded. Furthermore, trials with additional artefacts were rejected based on visual inspection. Only adults with at least 15 artefact-free trials in each condition were included in the analyses. Adults contributed a mean of 39.9 trials (20.4 in the Arm condition and 19.5 in the Leg condition). Only infants with at least 7 artefact-free trials were included in the analyses. Infants contributed a mean of 11.8 artefact-free trials to the analyses: 12.8 in the Arm condition (SD = 6.01; range: 7–27 trials) and 11.0 in the Leg condition (SD = 2.95; range: 7–17 trials).

EEG data was segmented into 4800 ms trials, consisting of a 1000 ms baseline and 3000 ms analysis period and a 400 ms buffer on either side of the segment. Time-frequency analyses were performed on each artefact-free trial by continuous wavelet transform using Morlet wavelets at 1 Hz intervals in the 5–25 Hz range. To eliminate distortion created by the wavelet transform, the first and last 400 ms of each trial were removed after the transformation. Activity in the alpha frequency range (8–13 Hz in adults, 6–9 Hz in infants) during 500 ms of the baseline period was subtracted from activity in the first 1500 ms of the analysis period. This 1500 ms period corresponded to the amount of time it took for the arm or leg in the video to make contact with the toy. Average wavelet coefficients were calculated for each participant by taking the mean across the trials.

#### 2.4.3 Frequency and channel selection

For the infant data, analyses focused on the 6–9 Hz frequency range. This frequency range was used because previous studies have demonstrated that the infant sensorimotor alpha rhythm is maximally suppressed at 7–8 Hz towards the end of the first year of life (Berchicci et al., 2011, Marshall et al., 2002, Stroganova and Orekhova, 2007) and to allow comparison with previous studies that used 6–9 Hz to investigate the infant sensorimotor alpha rhythm (e.g. Saby et al., 2013, Marshall et al., 2011, Virji-Babul et al., 2012). In the current study, the average maximally suppressed frequency was 7 Hz for the execution of arm movements, and 8 Hz for the execution of leg movements (see Table 1). This confirms that the 6–9 Hz frequency band encompasses those frequencies that are functionally related to the execution of actions in our sample. The analyses of the adult data focused on the 8–13 Hz alpha frequency range.

**Table 1:** The average maximally suppressed alpha frequency during the execution of arm and leg movements.

Participant number	Reaching (Hz)	Kicking (Hz)
02	8	6
04	7	7
08	8	6
09	7	7
10	6	8
11	9	8
12	6	6
14	8	9
19	6	8
22	10	10
24	6	7
25	6	9
26	7	10
31	6	7
33	6	6
35	8	7
41	9	10
44	8	9
45	8	7
49	7	6
50	7	8
52	7	7
54	7	8
55	7	9
**Average**	**7**	**8**

**Fig. 4:**
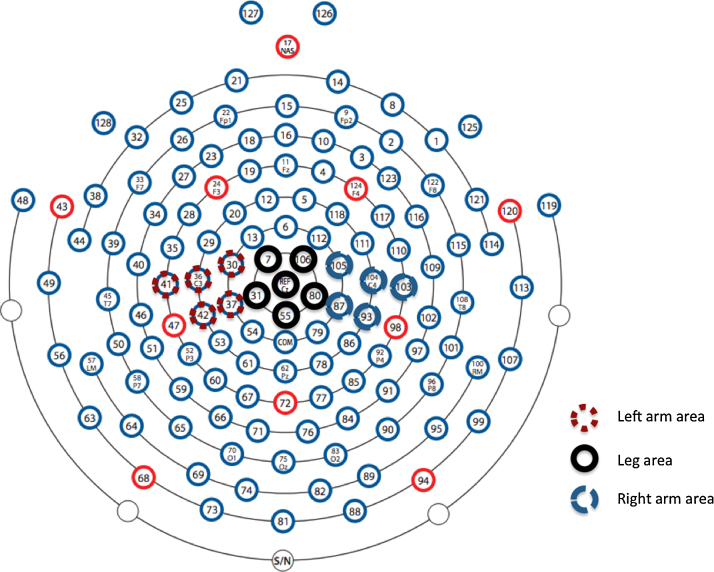
The Geodesic Sensor Net 128 channel lay-out in relation to the international 10–20 system of electrode placement. The three channel clusters located over the left arm area (electrodes 30, 36, 37, 41, 42), the leg area (electrodes 7, 31, 55, 80, 106, Cz), and the right arm area (electrodes 87, 93, 103, 104, 105) are marked.

Based on the previous studies investigating somatotopic organisation of sensorimotor alpha suppression during action execution in adults (Pfurtscheller et al., 1997, Pfurtscheller et al., 2000, Pfurtscheller et al., 2006) three clusters of channels located over the left lateral (electrodes 30, 36, 37, 41, 42), medial (electrodes 7, 31, 55, 80, 106, Cz), and right lateral (electrodes 87, 93, 103, 104, 105) sensorimotor cortex were selected. As can be seen in Fig. 4, the scalp locations of these left lateral, medial, and right lateral channel clusters correspond to the locations of C3, Cz, and C4 in the international 10–20 system of electrode placement. The medial cluster is thus located over the leg representation area of the sensorimotor cortex, while the left and right channel clusters are located over the bilateral arm representation areas (see also Fig. 1). For all analyses, activation over the left and right arm areas was initially averaged together into one lateral cluster to allow comparison of the results with the Saby et al. (2013) paper. In follow-up analyses, the bilateral arm cluster was split into left lateral, and right lateral channel positions. Suppression over these channel locations was analysed separately because: (1) even though infants occasionally used their left arm (15.4% of trials), or both arms (22.7% of trials), the majority of included reaches was performed with the right arm (61.9% of trials) and (2) previous studies investigating sensorimotor alpha suppression during action observation in infancy found the strongest activation over the left hemisphere (Southgate et al., 2009, Southgate et al., 2010, Southgate and Begus, 2013). Therefore, we expected that alpha suppression might be most clearly visible over the left lateral channel cluster. Note that because the leg representation area overlies the midline, splitting this channel cluster into a left and right leg cluster is not possible and therefore, the medial channel cluster represents the bilateral leg representation area in all analyses.

## 3 Results

### 3.1 Execution phase

**Fig. 5:**
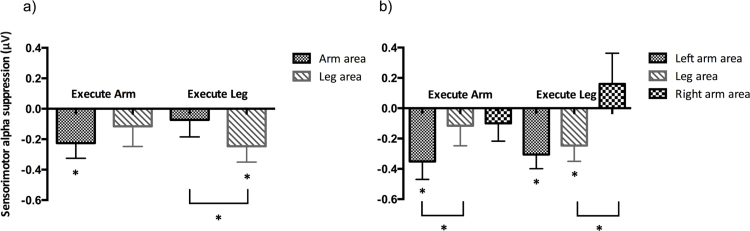
(a) Mean sensorimotor alpha suppression over the medial leg area and lateral arm areas during the execution of reaching and kicking actions. (b) Mean sensorimotor alpha suppression over the left arm area, the leg area, and the right arm area during the execution of reaching and kicking actions. Significant differences between conditions and significant suppression from baseline are indicated, *p < .05. Error bars represent 1 SEM.

A repeated-measures ANOVA on sensorimotor alpha suppression during action execution with effector (Arm vs. Leg) and location (Lateral vs. Medial channel positions) as within-subjects factors revealed the predicted interaction between effector and location, *F*(1, 23) = 6.661, *p* = .017, *η*_*p*_^2^ = .225. There were no significant main effects. Follow-up paired samples *t*-tests demonstrated that there was significantly more sensorimotor alpha suppression over the medial leg area than over the lateral arm areas when infants performed kicking actions, *t*(23) = −2.121, *p* = .045, while there was no significant difference between suppression over the arm and leg areas when infants performed reaching actions, *t*(23) = −1.057, *p* = .302 (see Fig. 5a for bar graphs of the mean sensorimotor alpha suppression). One-sample *t*-tests demonstrated that during the reaching actions only the suppression over the arm areas was significantly different from baseline, *t*(23) = −2.258, *p* = .034 while during the kicking actions only suppression over the leg area was significant, *t*(23) = −2.349, *p* = .028.

When sensorimotor alpha suppression over left and right arm areas was entered separately (rather than averaged together into one lateral arm cluster) into a repeated-measures ANOVA with effector (Arm vs. Leg) and location (Left Lateral vs. Medial vs. Right Lateral channel positions) as within-subjects factors, the interaction between effector and location was not significant, *F*(2, 46) = 1.831, *p* = .172, *η*_*p*_^2^ = .074. Planned comparisons demonstrated that there was significantly more sensorimotor alpha suppression over the left arm area than over the leg area during reaching, *t*(23) = −2.304, *p* = .031, and significantly more suppression over the leg area than the right arm area during kicking, *t*(23) = −2.693, *p* = .013 (see Fig. 5b for bar graphs of the mean sensorimotor alpha suppression and Supplementary Fig. 2 in Appendix B for topographical plots). One sample *t*-tests were performed to investigate which areas showed the strongest sensorimotor alpha suppression during the execution of reaching and kicking actions. During the execution of reaching actions, only the suppression over the left arm area was significantly different from baseline, *t*(23) = −2.965, *p* = .007, while during the execution of kicking actions there was significant suppression both over the left arm area, *t*(23) = −3.274, *p* = .003, and the leg area, *t*(23) = −2.349, *p* = .028.

### 3.2 Observation phase

#### 3.2.1 Adults

**Fig. 6:**
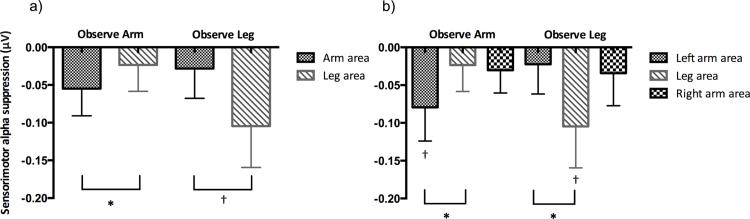
(a) Mean sensorimotor alpha suppression over the medial leg area and lateral arm areas during the observation of arm and leg movements in adult participants. (b) Mean sensorimotor alpha suppression over the left arm area, the leg area, and the right arm area during the observation of arm and leg movements in adult participants. Significant and marginally significant effects are indicated, *p < .05, ^†^.05 < *p* < .1. Error bars represent 1 SEM.

A repeated-measures ANOVA on sensorimotor alpha suppression during action observation with condition (Arm vs. Leg) and location (Lateral vs. Medial channel positions) as within-subjects factors revealed the predicted interaction between condition and location, *F*(1, 16) = 7.517, *p* = .014, *η*_*p*_^2^ = .320. There were no significant main effects. Follow-up paired samples *t*-tests demonstrated that there was significantly more sensorimotor alpha suppression over lateral arm areas than the medial leg area in the Arm condition, *t*(16) = −2.353, *p* = .032, and marginally significantly more suppression over the medial leg area than over the lateral arm areas in the Leg condition, *t*(16) = −2.077, *p* = .054 (see Fig. 6a for bar graphs of the mean sensorimotor alpha suppression).

When sensorimotor alpha suppression over left and right channel positions was entered separately (rather than averaged together into one lateral cluster) into a repeated-measures ANOVA with effector (Arm vs. Leg) and location (Left Lateral vs. Medial vs. Right Lateral channel positions) as within-subjects factors there also was a significant interaction between condition and location, *F*(2, 32) = 6.159, *p* = .005, *η*_*p*_^2^ = .278. Again there were no significant main effects. Follow-up paired samples t-tests demonstrated that there was significantly more sensorimotor alpha suppression over left lateral, than medial channels in the Arm condition, *t*(16) = −2.933, *p* = .010, and significantly more sensorimotor alpha suppression over medial than left lateral channels in the Leg condition, *t*(16) = −2.229, *p* = .041 (see Fig. 6b for bar graphs of the mean sensorimotor alpha suppression). There were no significant differences between medial and right channel positions for the Arm or Leg condition, all *p*'s > .099. We performed one-sample t-tests to investigate which areas showed the strongest sensorimotor alpha suppression in response to the observation of the pushing and kicking actions. In the Arm condition suppression was the strongest over the left arm area, *t*(16) = −1.791, *p* = .092, while in the Leg condition suppression was strongest over the medial leg area, *t*(16) = −1.900, *p* = .076.

#### 3.2.2 Infants

**Fig. 7:**
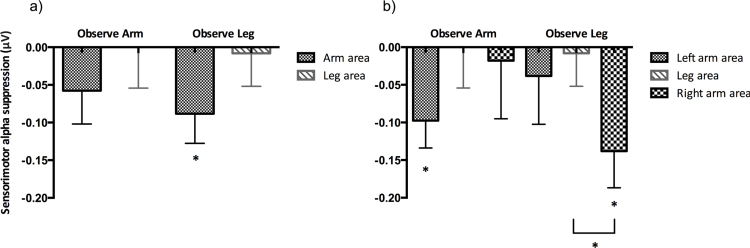
(a) Mean sensorimotor alpha suppression over the medial leg area and lateral arm areas during the observation of arm and leg movements in 12-month-old infants. (b) Mean sensorimotor alpha suppression over the left arm area, the leg area, and the right arm area during the observation of arm and leg movements in 12-month-old infants. Significant differences between conditions and significant suppression from baseline are indicated, *p < .05. Error bars represent 1 SEM.

A repeated measures ANOVA on sensorimotor alpha suppression during action observation with location (Lateral vs. Medial channel positions) as within subjects factor, and condition (Arm vs. Leg) as between subjects factor, demonstrated no effect of condition, *F*(1, 25) = .149, *p* = .703, *η*_*p*_^2^ = .006, a trend towards an effect of location, *F*(1, 25) = 3.540, *p* = .072, *η*_*p*_^2^ = .124, and no interaction between location and condition, *F*(1, 25) = .069, *p* = .795, *η*_*p*_^2^ = .003. Planned comparisons demonstrated that there were no significant differences between sensorimotor alpha suppression over lateral arm areas and the medial leg area in the Arm condition, *t*(11) = 1.191, *p* = .259, or in the Leg condition, *t*(14) = 1.511, *p* = .153 (see Fig. 7a for bar graphs of the mean sensorimotor alpha suppression). One-sample t-tests demonstrated that only in the Leg condition was the suppression over the lateral arm areas significantly different from baseline, *t*(14) = −2.236, *p* = .042.

A repeated measures ANOVA with sensorimotor alpha suppression over left lateral, medial, and right lateral channel positions also demonstrated no significant main effects or interactions, all *p*'s > .225. One sample *t*-tests were performed to investigate which areas showed the strongest sensorimotor alpha suppression when infants observed arm and leg actions. These analyses showed that infants in the Arm condition demonstrated significant suppression over the left arm area, *t*(11) = −2.691, *p* = .021, while infants in the Leg condition demonstrated significant alpha suppression over the right arm area, *t*(14) = −2.838, *p* = .013. This suppression over the right arm area in the Leg condition was significantly greater than the suppression over the leg area, *t*(14) = 2.444, *p* = .028 (see Fig. 7b for bar graphs of the mean sensorimotor alpha suppression and Supplementary Fig. 3 in Appendix B for topographical plots).

## 4 Discussion

The current study investigated whether sensorimotor cortex activation during action execution and observation is somatotopically organised in infancy. We measured sensorimotor alpha suppression at channel positions located over the arm and leg areas during the execution and observation of arm and leg actions. Notwithstanding the limitations of using surface electrodes for cortical localisation, the activation patterns that were found during action execution were consistent with the known somatotopic organisation of the sensorimotor cortex (Penfield and Rasmussen, 1950). Specifically, when 12-month-old infants performed reaching actions there was more sensorimotor alpha suppression over the arm areas than over the leg areas, and when they performed kicking actions there was more suppression over the leg areas than over the arm areas. This somatotopically-organised sensorimotor cortex activation during the execution of arm and leg movements is consistent with previous EEG studies with adults (Pfurtscheller et al., 1997, Pfurtscheller et al., 2000, Pfurtscheller et al., 2006) and with studies suggesting that the somatotopic organisation of the cortex develops early in life (Milh et al., 2007).

Although the sensorimotor alpha suppression was only significantly different from baseline over the left arm area when infants performed reaching actions, there was significant suppression over both the left arm area and the medial leg area when infants performed kicking actions. One possible explanation for this finding is that even though all kicking trials in which infants made overt arm movements were excluded from analysis, infants might still have been using their arms to maintain balance on their parents lap while moving their legs. Alternatively, as the motor system is thought to be involved in any kind of event prediction (Schubotz, 2007, Schubotz and von Cramon, 2002, Schubotz and von Cramon, 2004, Wolfensteller et al., 2007) and sensorimotor alpha suppression over the left hemisphere has been related to predictive processes in infancy (Southgate et al., 2009, Southgate et al., 2010, Southgate and Begus, 2013), the sensorimotor alpha suppression over the left arm area during the execution of the kicking actions may reflect infants’ prediction of the movement of the mobile. As a result of the suppression over the left arm area during the execution of both reaching and kicking actions, the presence or absence of suppression over the medial channel cluster overlying the leg area most clearly distinguishes between the two actions. This result validates the use of sensorimotor alpha suppression over the medial channel cluster as a functional index of leg area activation in the sensorimotor cortex.

The second aim of the study was to investigate whether sensorimotor alpha suppression during action observation is also somatotopically-organised. We found that adults activated their sensorimotor cortex in a clear somatotopic fashion during the observation of arm and leg actions. When they observed arm actions there was more suppression over the arm areas than over the leg area, and when they observed leg actions the opposite pattern of activation was found. This finding is consistent with previous fMRI studies demonstrating that motor cortex activation is somatopically-organised during action observation (e.g. Buccino et al., 2001), and confirms that our stimuli were able to elicit somatotopically-organised sensorimotor alpha suppression in adult participants.

Based on the results from the adult participants, and those of another study with slightly older infants (Saby et al., 2013), we expected to find similarly somatotopically-organised activation patterns in the 12-month-old infants. However, we did not find evidence for this. There was no effect of condition, and the expected within-condition differences, with more suppression over the lateral compared to the medial clusters in the Arm condition and the opposite pattern in the Leg condition, were absent. This absence of a somatotopic organisation of sensorimotor alpha suppression during the observation of arm and leg actions is inconsistent with previous findings with 14-month-olds (Saby et al., 2013). A possible explanation for this finding is that in the study by Saby et al. (2013) the actions were presented in a live interaction with the experimenter, while in the current study the actions were presented on video. It has been shown that actions observed in live settings elicit greater sensorimotor cortex activation than actions presented on video (Ruysschaert et al., 2013, Shimada and Hiraki, 2006). However, although this may have weakened overall activation in the current study, it is unlikely to be the cause for the absence of a somatotopic effect as significant activation from baseline was found in both conditions and has been demonstrated in several previous studies that used video stimuli (e.g. Southgate and Begus, 2013, Southgate and Vernetti, 2014). Alternatively, as the videos only showed the effector without the body of the actor, it may have been difficult for the infants to recognise the effector performing the action. To our knowledge there is no study that directly compared sensorimotor alpha suppression in response to actions performed by an actor whose full body is visible compared to actions performed by ‘disembodied’ limbs. Although previous sensorimotor alpha studies have successfully used stimuli in which only a part of the actor's body was visible (de Klerk et al., 2015, Southgate et al., 2009, Southgate et al., 2010, Southgate and Begus, 2013), it is possible that seeing the actor's whole body is particularly important to obtain somatotopic effects, and future studies are needed to investigate this possibility.

When we looked at the areas of the sensorimotor cortex that were significantly activated during action observation, we found that both infants in the Arm and the Leg condition showed activation over the arm areas. Specifically, infants in the Arm condition showed significant suppression over the left arm area, and infants in the Leg condition showed significant suppression over the right arm area (and this suppression was significantly stronger than that measured over the leg area). There was no significant suppression over the leg areas in either of the conditions. The surprising finding that infants activated the arm, and not the leg area during the observation of the kicking actions, seems to be consistent with the idea that infants might have been *emulating* the goal of the action, i.e. reproducing the outcome of the action using their own means (Tomasello, 1996). Actions are organised in a hierarchical manner, with the overarching goal of an action being represented at the highest level of the hierarchy, followed by the motor signals that lead to the muscle activation through which the goal can be achieved, and with the kinematic level, which describes the configuration of the movements needed to be performed, at the lowest level of the hierarchy (Hamilton and Grafton, 2007). It has been suggested that observed actions are first interpreted at the highest possible level of this action hierarchy before they are passed on to the motor system, supporting the generation of predictions about how the action will unfold (Csibra, 2007, Wilson and Knoblich, 2005). Considering that there are many different ways in which a goal or subgoal can be achieved, it follows from this *emulative action reconstruction* account that if goal attribution is possible, even if infants have little experience with the action they are observing, they may emulate the outcome in an alternative way. It has been shown that infants from as young as 6 months of age can interpret unfamiliar actions as goal-directed as long as the action results in a (salient) change of state in the environment (Jovanovic et al., 2007, Kiraly et al., 2003). While infants observe grasping and pushing actions many times a day, kicking actions are much less frequently observed. Thus, infants in the current study may have been emulating how they would displace the toy themselves when they were watching the relatively unfamiliar kicking actions, leading to activation of the arm, rather than the leg areas. Although speculative, this interpretation of the data is consistent with several recent studies demonstrating that when adult participants were presented with actions performed with an unusual effector, e.g. grasping actions performed by the foot instead of the hand, parts of the motor system were more sensitive to the action goal than to the effector that was performing the action (Jastorff et al., 2013, Lorey et al., 2014, Rijntjes et al., 1999, Senna et al., 2014), suggesting that participants were emulating how they would perform the action rather than simply ‘mirroring’ the activation of the effector they were observing. Similarly, Gazzola et al. (2007) found that when individuals with congenital aplasia of the upper limbs were presented with grasping actions, they showed activation of the foot or mouth areas that they would typically use to achieve the same goal. The results of the current study suggest that infants might also emulate, rather than mirror, observed actions when the actions are relatively unfamiliar to them. Nevertheless, such an interpretation of the data is speculative at this stage and future studies will be needed to investigate more systematically whether sensorimotor cortex activation during action observation in infancy is goal-, or effector-specific.

It is unclear why infants in the Arm condition showed suppression over the left arm area, while infants in the Leg condition showed suppression over the right arm area. One possible explanation could be that infants in the Leg condition were predominantly left-handed while infants in the Arm condition where predominantly right-handed. However, there were no significant differences in handedness between the two groups, *X*^2^ (1, *N* = 20) = 1.485, *p* = .223, as assessed by the proportion of left-handed vs. right-handed reaches during the execution phase of the study. Alternatively, the difference in lateralisation between the Arm and Leg condition could be due to difference in the proportion of left- and right-effector actions that was observed. Indeed, even though left-ward and right-ward stimuli were presented randomly, by chance more trials with leftward movements (performed by the right leg) were included in the Leg condition, and more trials with rightward actions (performed by the left arm) where included in the Arm condition, *F*(1,25) = 3.892, *p* = .060. This interpretation is consistent with previous studies that suggest that when actions are observed from a third-person perspective, there is more activation over the ipsilateral motor cortex, i.e. observing a right-handed action increases activity in the right hemisphere (e.g. Shmuelof and Zohary, 2008, Vingerhoets et al., 2012).

## 5 Conclusions

This study demonstrated that 12-month-old infants, like adults, show somatotopically organised sensorimotor cortex activation during action execution. Adults also showed somatotopically-organised activation when they observed goal-directed arm and leg actions. In contrast, infants did not show somatotopically organised activation during action observation, but instead activated the arm areas when observing both arm and leg actions. These findings suggest that infants might have activated the motor programme of the effector that they more commonly use to displace objects (i.e. the arm area) during the observation of the kicking actions. Thus, although the somatotopic arrangement of the sensorimotor cortex for *action execution* seems to be in place in the first year of life, the somatotopic organisation of sensorimotor cortex activation during *action observation* may depend on infants’ understanding of the action goal and their expectations about how this goal will be achieved. Future studies are needed to investigate whether infants indeed flexibly employ their motor system during action observation to generate predictions about how the action will unfold.

## Conflict of interest

The authors declare that they have no conflict of interest.

## Appendix A Supplementary data

See Appendix A for topographical plots of example execution trials from a representative infant participant. See Appendix B for topographical plots depicting the scalp distribution of sensorimotor alpha suppression during the execution and observation of arm and leg movements for the infant participants.
