# Supplementary material for: An EEG study on the somatotopic organisation of sensorimotor cortex activation during action execution and observation in infancy
Source: Dev Cogn Neurosci. 2015 Aug 17;15:1–10. doi: 10.1016/j.dcn.2015.08.004 (PMC4649773; doi:10.1016/j.dcn.2015.08.004)
Supplement: Supplementary file 1 [file mmc1.pdf]

Appendix A

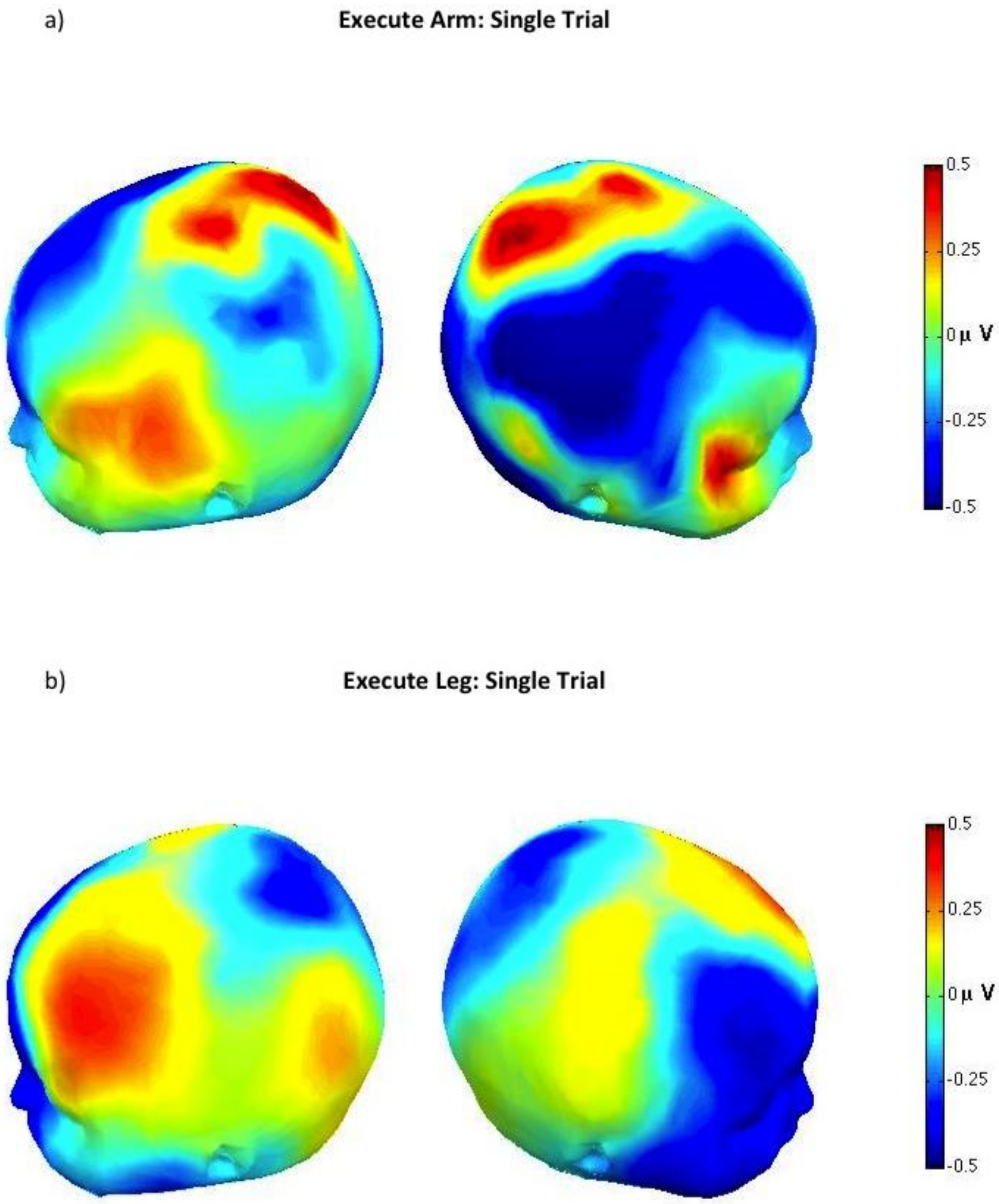

Supplementary Figure 1. Topographical plots demonstrating the scalp distribution of sensorimotor alpha suppression (6-9 Hz during the execution of a) a reaching action and b) a kicking action. The plots show baseline-corrected activity from one trial of a representative infant participant (participant 19).

Appendix B

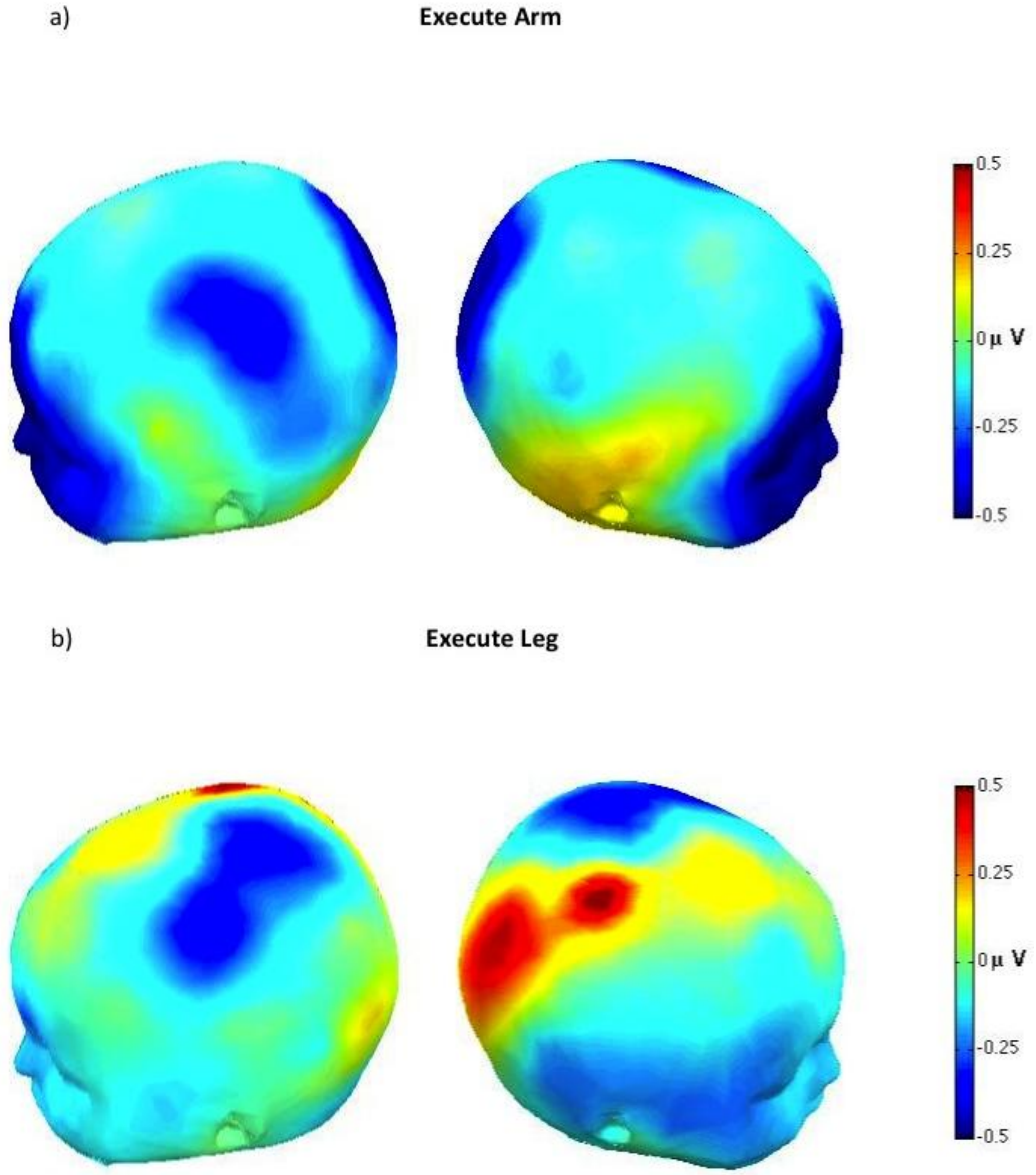

Supplementary Figure 2. Topographical plots demonstrating the scalp distribution of sensorimotor alpha suppression (6-9 Hz) in infant participants during the execution of a) arm actions and b) leg actions.

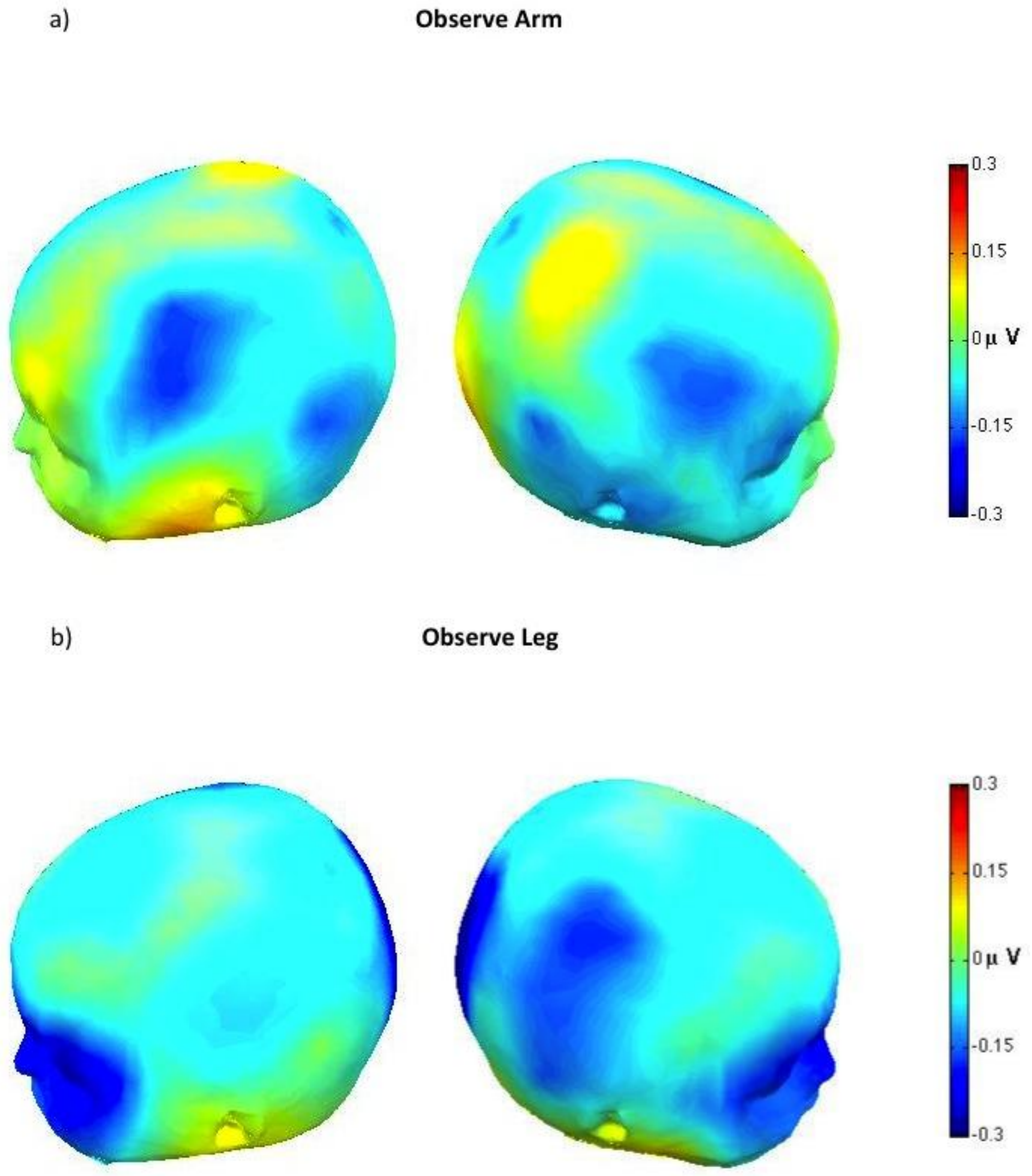

Supplementary Figure 3. Topographical plots demonstrating the scalp distribution of sensorimotor alpha suppression (6-9 Hz) in infant participants during the observation of a) arm actions and b) leg actions.
